# Neonatal chest image quality addressed through training to enhance radiographer awareness

**DOI:** 10.4102/hsag.v23i0.1067

**Published:** 2018-03-26

**Authors:** Hesta Friedrich-Nel, Belinda van der Merwe, Beatrix Kotzé

**Affiliations:** 1 Faculty of Health and Environmental Sciences, Central University of Technology, South Africa

## Abstract

**Background:**

Diagnostic radiographers working in the neonatal intensive care unit primarily aim to produce an image of optimal quality using optimal exposure techniques without repeating exposures, to keep neonatal radiation dose to a minimum.

**Objectives:**

The aim of the study was to determine whether radiographers were producing optimal quality chest images and, if not, whether additional training could contribute to reaching this goal in the Free State Province of South Africa.

**Methods:**

Neonatal chest image quality was determined in the Neonatal Intensive Care Unit by using a checklist based on and compiled from published guidelines to evaluate the quality of 450 randomly-selected images. Thereafter, a training programme was designed, based on the evaluation criteria of the checklist and image quality areas identified. The training also referred to positioning techniques that should be applied to ensure optimal image quality. After presentation of the training, 450 newly-produced neonatal chest images were evaluated. These images were selected through purposive sampling as this evaluation only included images of participating radiographers who completed the training.

**Results:**

Image quality that showed significant improvement included a reduction in electrocardiogram lines superimposed on chest anatomy, a tendency to centre closer to thoracic vertebra four, and visible four-sided collimation on images. Image quality areas with no significant enhancement were the absence of lead markers and radiation shielding.

**Conclusion:**

The study has shown that a training programme has the potential to improve neonatal chest image quality.

## Introduction

Patient diagnosis that incorporates diagnostic radiography requires the delivery of images of the highest possible quality by diagnostic radiographers. However, an important prerequisite is to keep the radiation dose to all patients, including neonatal patients, as low as possible in accordance with the ALARA (as low as reasonably achievable) principle (Strauss & Kaste 2006). The International Atomic Energy Agency, in close collaboration with the World Health Organization, gives special attention in its recommendations to the restriction of radiological diagnostic procedures on children (International Atomic Energy Agency 2002). Pedrosa de Azevedo, Osibote and Boechat (2006) emphasise that if a neonatal examination should be performed, the use of special lead shielding devices and correct techniques are compulsory.

In a radiology practice located in a private hospital in the Free State Province of South Africa, the practising radiologists questioned whether the radiographers working in the Neonatal Intensive Care Unit (NICU) were producing images of the highest quality and providing optimal radiation protection for the neonates during mobile radiography. A similar problem was recorded by an US-based study (Hellwig & Wilson 2013). The problem statement was determined after the imaging department’s management staff noticed an increase in ‘babygram’ images that showed minimum collimation without radiation shielding in place.

## Literature perspective

Similar challenges arising from paediatric and neonatal imaging practices have led to the Alliance for Radiation Safety in Pediatric Imaging, also known as the Image Gently campaign (Image Gently 2014), which promotes additional radiography training programmes with the aim to encourage changes in these imaging practices. This alliance promotes additional training to ensure that patients receive the right optimal imaging examination, at the correct time, with the minimum radiation dose (Goske et al. 2011; Willis 2009).

The Pan African Congress of Radiology and Imaging, along with other organisations representing radiation health workers, launched the AFROSAFE campaign in 2015 with the goal to identify and address medical radiation protection concerns in Africa. The stimulus for AFROSAFE was the Bonn Call-for-Action (2013), which is a joint statement by the World Health Organization and the International Atomic Energy Agency (International Atomic Energy Agency 2015).

The Bonn Call-for-Action (2013) is aimed at: (1) strengthening the application of radiation protection for patients; (2) attaining the highest benefit-to-risk ratio for all patients through appropriate medical radiation utilisation; (3) ensuring the integration of radiation protection throughout the health care system; (4) increasing patient awareness by means of discussions with patients about benefit-to-risk ratios; and (5) enhancing the safety of quality radiological procedures in medicine. In order to achieve these aims, the Bonn Call-for-Action (2013) encourages health care workers to take action during the next decade by participating in these 10 main actions. Two of these actions relate directly to the scope of this article; firstly, it requires healthcare workers to promote putting into practice the optimisation principle with regard to protection and safety when working with children. Secondly, health care workers are also called upon to strengthen education and training relevant to radiation protection. The second action involves further professional development through continuous training opportunities.

Governmental regulations and codes (Republic of South Africa Department of Health 1973, 1974) reflect the emphasis of international and local health bodies on the correct imaging of paediatric patients, including neonatal patients. Imaging of paediatric patients should be performed only when justified. Should such an examination be justified, it should be completed with the correct equipment to ensure the lowest possible radiation dose to the patient, but with the production of an optimal image to ensure optimisation of the examination.

## Purpose

The purpose of this study was to address the image quality of mobile neonatal chest images executed in a NICU by means of a training programme to increase awareness among radiographers, and consequently, deliver optimal radiation protection measures to neonates.

## Research method and design

An evaluation study was performed on neonatal chest images produced in the NICU of three hospitals in the Free State Province over a period of 11 months. These hospitals have NICUs and radiological imaging departments that use computed radiography systems for the imaging of neonates. The quality of 450 randomly-selected chest images produced in the NICU prior to the delivery of a training programme was assessed by means of a piloted checklist based on and compiled from guidelines in image quality literature, such as those proposed by Pedrosa de Azevedo et al. (2006). This checklist was furthermore based on different criteria for neonatal chest image quality reflected in criteria specified by international boards such as the European Commission, and the evaluation criteria described by authors such as Bontrager and Lampignano (2014) and McQuillen Martensen (2011). The design of the checklist entailed a complex process that considered various aspects, namely, (1) correct centring of the main radiation beam; (2) incorrect rotation of the neonatal thorax; (3) correct main beam angulation; (4) artefacts included on chest anatomy; (5) mandatory lead marker placement; (6) required lead shielding visibility; and (7) optimal collimation to the chest area of interest.

After the initial checklist investigation, the training programme was presented to 56 qualified diagnostic radiographers practising in the participating hospitals, rotating through the NICU. The training programme was based on the evaluation criteria of the checklist used for the initial assessment of the images. The training was also informed by areas of image quality in need of improvement as identified by the initial checklist investigation and also included recently updated radiographic positioning techniques described in literature (Bontrager & Lampignano 2014; McQuillen Martensen 2011).

On completion of the training programme, the quality of 450 neonatal chest images produced by the radiographers who completed the training (purposively sampled), was re-evaluated in the NICU to establish whether image quality improved after delivery of the training programme, as called for by the Bonn Call-for-Action (2013) and advised by the Image Gently (2014) campaign. The checklist used for the initial assessment of the images was used for the re-assessment as well.

### Ethical considerations

Co-operation and consent were obtained from the managerial bodies of each hospital. Neonatal chest images were assessed but no personal information of the neonates was recorded. Only neonatal chest images produced in the NICU for diagnostic purposes were included in the study. The identity of participating radiographers was protected and participation was voluntary. Radiographers working in the participating institutions were invited to attend an information session; those interested in participating provided informed consent after this session. Ethical approval to conduct the study was obtained from the Ethics Committee of the Faculty of Health Sciences at the University of the Free State in Bloemfontein, South Africa (ECUFS No. 163/2011).

## Analyses

Descriptive statistics, namely, frequencies and percentages for categorical data, and means and standard deviations (or medians and percentiles for continuous data), were calculated. The descriptive statistics were calculated separately for pre- and post-training for the hospitals. Median values were compared using either the chi-squared test for differences between frequencies or the Kruskal–Wallis test for differences between medians.

## Results

The pre-training evaluation showed areas in which the image quality could be addressed (Table 1). Problems with regard to radiographic positioning included inappropriate centring (64.9%) and artefacts superimposing chest anatomy (56.4%), with the most observed artefact being electrocardiogram lines. Other observations involved the absence of lead shielding visibility, which should be applied over the pelvis (98.7%), and a lack of collimation (74.9%), resulting in the inclusion of anatomical structures not required for diagnosis.

**TABLE 1:** Comparison between pre- and post-training results.

Evaluation criteria on checklist	Percentages (*n* = 450)		Comparison *p*
	Pre-training	Post-training	
Inappropriate centring of the main radiation beam	64.9	60.0	0.13
Rotation of the neonatal thorax	56.7	61.3	0.20
Inadequate main beam angulation	41.6	42.7	0.74
Included artefacts on chest anatomy:	56.4	55.8	0.84
ECG lines	61.9	41.8	0.001
Mandatory lead marker absent	66.4	63.1	0.23
Required lead shielding NOT visibility	98.7	98.9	0.76
Lack in optimal collimation to the chest area	74.9	65.3	0.002

Note: NOT denotes the required anatomical market to indicate the anatomical side of a neonate was absent (not visible) on the image.

At the end of the training programme, participating radiographers completed an evaluation form to share their perceptions of the educational properties of the training sessions. The results showed in Table 2 indicate that the perception of the radiographers who attended the sessions was that they benefited from the training programme.

**TABLE 2:** Participants’ perception of the training programme.

Perception of participants	Percentages (*n* = 56)
Usefulness of content	96.4
Presenter effectiveness	89.2
Opportunity to interact	91.1

After the training programme had been completed and the radiographers’ perceptions recorded, the image quality of neonatal chest images was re-evaluated, as shown in Table 1. The significance in changes noted was calculated by comparing the results of pre- and post-training and is displayed as *p*-values.

## Discussion

The radiographic positioning techniques visible on images did not show a significant improvement, with *p*-values ≥ 0.05. The centring point did not show a significant change (64.9% of images were incorrectly centred before training and 60.0% afterwards), but the overall trend was observed to be closer to the prescribed fourth thoracic vertebra (T4) (Bontrager & Lampignano 2014; McQuillen Martensen 2011). Correct centring is essential, because it limits geometric unsharpness and image distortion (Carlton & Adler 2014; McQuillen Martensen 2011).

The images produced after the training programme revealed incorrect rotation in 61.3% of images (compared to 56.7% pre-training). A lack of angulation of the main radiation beam was still present in 42.7% of images (vs. 41.6% of pre-training evaluation) and artefacts were superimposed on chest anatomy in 55.8% of images (compared to 56.4% of first evaluation). However, the most frequently observed artefact, the electrocardiogram lines artefact, did show a significant decrease (*p* = 0.001) from pre- to post-training. This artefact was observed at the highest frequency of all the artefacts recorded; therefore, a reduction in the observation of this artefact specifically implies a significant enhancement of image quality.

A serious concern was the lack of the lead marker placement and the absence of lead shielding evident on images both before and after the training programme was conducted. During pre-training, 66.4% of images did not present with lead markers and 98.7% images did not include visible lead shielding. After the training programme, 63.1% of images presented without lead markers and 98.9% of images showed no visibility of lead shielding. The evaluations before and after the training indicated that lead markers were not visible on an average of 64.8% of images and lead shielding on 98.9% of images. Both these aspects are addressed by the South African Department of Health (Republic of South Africa Department of Health 1973, 1974) in the Acts of 1971 and 1974, respectively, with regard to regulations concerning the control of electronic products and the scope of radiography profession. Lead markers and shielding cannot be considered as optional but are imperative and non-negotiable.

The amount of collimation not found on images decreased significantly (*p* = 0.002) from pre-training (74.9%) to post-training (65.3%). In addition, most images included the relevant anatomy, and during the post-training evaluation, a decrease in the inclusion of additional anatomy laterally (*p* = 0.02) and inferiorly (*p* = 0.01) was noted. This observation indicates that sensitive structures, such as the humerus (included in 63.4% of images during pre-training vs. 51.3% of images in post-training) and pelvis, were more frequently excluded after the training programme because of improved levels of collimation. The pelvis was included on 30.6% of images before the programme versus 20% of images showing the pelvis after the programme. In addition, radiographers were centring closer to the fourth thoracic vertebra (T4), which also led to the exclusion of irrelevant anatomy.

Furthermore, of particular interest in this study was the fact that the clinical history was absent from referral letters in more than 90% of cases (94% pre-training and 96.7% post-training). Clinical history is important, because based on the clinical information provided, radiographers determine anatomical structures to be included, radiographic techniques and radiation parameters (Morris 2003). Without a clinical history, the justification for neonatal radiographic examination is compromised. Radiation regulations (Republic of South Africa Department of Health 1973, 1974) require this history, and it should therefore be included.

## Trustworthiness

The checklist was valid and dependable because its design was based on literature and it was benchmarked for research studies that used a similar research instrument (Bontrager & Lampignano 2014; McQuillen Martensen 2011; Pedrosa de Azevedo et al. 2006). The checklist therefore measured what it was supposed to measure. In addition, the checklist was piloted in a similar population group as that involved in the study. The construction of the checklist was of such a nature that it delivered uniform results time after time, because it consisted mainly of structured image quality areas.

The training programme was considered to be valid and reliable because its design was based on literature relating to neonatal chest image quality, and on updated positioning techniques that can ensure optimal neonatal chest image quality (Carlton & Adler 2014; McQuillen Martensen 2011). Data from the pre-training evaluation of the study confirmed the inclusion of certain literature areas in the training programme.

## Limitations and recommendations

The most notable limitation of this study was the design and presentation of the training programme, which was a single-person effort. The training programme did not involve assessment that evaluated the educational value of the programme. The implementation of a neonatal image quality audit programme by imaging departments that service NICUs is recommended. Such an audit programme will align with the goal of the Image Gently (2014) campaign – to change practice – and the Bonn Call-for-Action (2013) to enhance optimisation and educational opportunities pertaining to radiation protection. The checklist has proven to be a useful instrument to discern areas of improvement in terms of image quality. The areas of image quality that caused concern could be identified with this instrument. The checklist can be utilised as part of a neonatal image quality audit programme.

## Conclusion

An aspect of neonatal chest image quality that improved after the training programme was the level of collimation observed, which lowers the radiation dose to the neonate. In addition, after the programme, images were centred closer to the fourth thoracic vertebra which excludes anatomical areas not of interest and sensitive to radiation. Electrocardiogram lines artefacts, which were a common phenomenon before the training programme, were reduced significantly. However, image quality areas that did not improve following the programme included rotation of the thorax, angulation of the main radiation beam and the absence of lead shielding and lead markers. The research has shown that a training programme has the potential to improve neonatal chest image quality, which aligns well with the main concerns of the Image Gently (2014) campaign, namely to change practice.
